# The complete mitochondrial genome sequence of *Aquilaria sinensis*

**DOI:** 10.1080/23802359.2020.1869609

**Published:** 2021-02-08

**Authors:** Zheng-Feng Wang, Hong-Lin Cao

**Affiliations:** a Key Laboratory of Vegetation Restoration and Management of Degraded Ecosystems, South China Botanical Garden, Chinese Academy of Sciences, Guangzhou, China; b Center for Plant Ecology, Core Botanical Gardens, Chinese Academy of Sciences, Guangzhou, China; c Southern Marine Science and Engineering Guangdong Laboratory (Guangzhou), Guangzhou, China

**Keywords:** *Aquilaria sinensis*, genome assembly, high-throughput sequencing, mitochondrion

## Abstract

*Aquilaria sinensis*, endemic to China, is an economically important evergreen tree species and a source of agarwood. Due to the high market demand for agarwood, this species is heavily overexploited in the wild and is now listed as an endangered species. Although its nuclear and chloroplast genomes have been previously reported, little is known about its mitochondrial genome. Using the paired-end short reads generated by the Illumina sequencing platform, we assembled and herein report the mitochondrial genome of *A*. *sinensis* for future phylogenetic, evolutionary, and preservative studies. The length of the *A*. *sinensis* mitochondrial genome was found to be 341,829 bp and the GC content was 45.01%. A total of 32 protein-coding genes, 19 tRNA genes, and three rRNA genes were annotated. The phylogenetic tree indicated that *A*. *sinensis* is most closely genetically related to *Vigna radiata*.

*Aquilaria* (Thymelaeaceae) species are evergreen trees that are distributed from South to Southeast Asia. These trees are best known for the production of agarwood, which is a resinous heartwood used in traditional medicine, incense, and perfume, as well as for decorations and art pieces. There are 21 species in this genus, 13 of which are able to produce agarwood (Wang et al. 2020). An agarwood is produced in the trunk and main branches as a result of wounding, such as by worm, microbial, or fungal infections, and is harvested by felling or cutting down the tree. Therefore, natural agarwood is becoming more rare due to illegal logging. All species of *Aquilaria* are currently listed as vulnerable to critically endangered on the International Union for Conservation of Nature (IUCN) Red List. *Aquilaria sinensis* (Lour.) Spreng is endemic to South China. It is the only species to produce agarwood used as a crude medicine in China (Wang et al. 2020). Although its nuclear and chloroplast genomes have been previously reported (Wang et al. 2016, 2020), no complete mitochondrial genomic resources are currently available.

The study of plant mitochondria has a long history. The first research dates back to 1904 (Ernster and Schatz 1981), with the first plant mitochondrial genome reported in 1992 (Oda et al. 1992) for *Marchantia polymorpha*. In recent decades, with the development of next generation sequencing technologies, whole genomes, including nuclear and organelle genomes in variable organisms, were able to be fully sequenced and assembled (Wang et al. 2016, 2020; Chen et al. 2020; Ding et al. 2020; Kim et al. 2020; Liu et al. 2020; Miao et al. 2020; Shao et al. 2020; Yang et al. 2020). However, because plant mitochondrial genomes are much more variable in structure and size (Mackenzie and McIntosh 1999) than chloroplast genomes, fewer plant mitochondrial genomes have been assembled and reported compared to chloroplast genomes and their animal counterparts. In plants, the mitochondrion is an important organelle involved in respiration and metabolism (Mackenzie and McIntosh 1999). Therefore, in this study, we report the complete mitochondrial genomic sequence of *A*. *sinensis* to provide a resource for future effective conservation and evolutionary studies.

Leaf samples were collected from Dianbai County, China (21°38′27.21″N, 111°15′14.52″E) and a voucher specimen was deposited in the Herbarium of the South China Botanical Garden (no. IBSC-T-201709Y14). From this sample, approximately 160 Gb whole genome sequencing reads were generated with the Illumina HiSeq X Ten (Illumina, San Diego, CA) platform using the paired-end (2 × 150 bp) strategy. Using 14 previously reported mitochondrial genomes (Figure 1) as references, reads related to mitochondrial sequences were extracted using GetOrganelle 1.7.1 (Jin et al. 2020). The extracted reads were used to assemble the mitochondrial genome by NOVOPlasty 4.2.1 (Dierckxsens et al. 2017) using the parameter ‘mito_plant’. After assembly, the genome was polished by NextPolish 1.3.0 (Hu et al. 2020) and then annotated by GeSeq (Tillich et al. 2017) and PGA (Qu et al. 2019). The annotated mitochondrial genome was deposited in GenBank with the accession number MW057771. Microsatellites across the mitochondrial genome were determined by MISA-web (Sebastian et al. 2017) using its default parameter setting. The phylogenetic tree for *A*. *sinensis* and another 14 species of the Malvales order (Figure 1) was generated using mashtree v1.2.0 (Katz et al. 2019). For those genes shared by *A*. *sinensis* and another species, the nucleotide diversity (*Pi*) value was calculated using MEGA-X v10.1.8 (Kumar et al. 2018).

**Figure 1.**
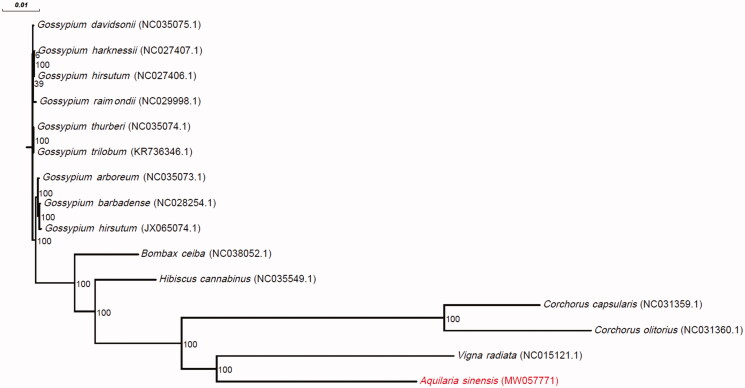
Phylogenetic tree from the complete mitochondrial genome sequences of *Aquilaria sinensis* and 14 additional species. GenBank accession numbers are denoted in parentheses. Bootstrap percentages (1000 replicates) are shown at nodes.

The length of the complete mitochondrial genome of *A*. *sinensis* was 341,829 bp. The base composition of the mitochondrial genome was 27.45% T, 27.54% A, 22.44% C, and 22.57% G, with a GC content of 45.01%. The base composition of the *A*. *sinensis* mitochondrial genome was similar to the other 14 Malvales species (Table S1), with the GC content ranging from 42.85% to 45.11%. Annotation analysis revealed a total of 32 protein-coding genes, 19 transfer RNA genes, and three ribosomal RNA genes in the *A*. *sinensis* mitochondrial genome. The length of protein-coding genes ranged from 81 to 2826 bp, with a mean of 896.938 bp, the exon length ranged from 81 to 1968 bp, with a mean of 720.968 bp, and the intron length ranged from 777 to 1389 bp, with a mean of 1035.667 bp (Table S2). Compared to the other species, *A*. *sinensis* had the shortest mean gene, exon, and intron lengths. A total of 44 microsatellites were identified in the *A*. *sinensis* mitochondrial genome (Table S3). Among them, the majority were mononucleotide-repeat microsatellites (79.54%). There were only seven protein-coding genes shared between *A*. *sinensis* and the other species studied. The *Pi* values ranged from 0.009437 for the ccmB gene to 0.076923 for the rpl16 gene (Table S4). The phylogenetic tree showed that the mitochondrial genome of *A*. *sinensis* was closely related to that of *Vigna radiata* (Figure 1).

## Data Availability

The complete mitochondria genome sequences of *Aquilaria sinensis* have been deposited in GenBank under the accession numbers MW057771 and is also accessible at https://doi.org/10.13140/RG.2.2.27460.65925. The associated BioProject, SRA, and Bio-Sample numbers are PRJNA532686, SRR8892930, and SAMN11412194, respectively.
